# Effect of Different Extraction Methods on the Quality and Biochemical Attributes of Pomegranate Juice and the Application of Fourier Transformed Infrared Spectroscopy in Discriminating Between Different Extraction Methods

**DOI:** 10.3389/fpls.2021.702575

**Published:** 2021-08-23

**Authors:** Ebrahiema Arendse, Helene Nieuwoudt, Olaniyi Amos Fawole, Umezuruike Linus Opara

**Affiliations:** ^1^SARChI Postharvest Technology Research Laboratory, Faculty of AgriSciences, Africa Institute for Postharvest Technology, Stellenbosch University, Stellenbosch, South Africa; ^2^Department Viticulture and Oenology, Institute for Wine Biotechnology, Stellenbosch University, Stellenbosch, South Africa; ^3^Postharvest Research Laboratory, Department of Botany and Plant Biotechnology, University of Johannesburg, Johannesburg, South Africa; ^4^UNESCO International Centre for Biotechnology, Nsukka, Nigeria

**Keywords:** *Punica granatum* L, antioxidant activity, organic acids, chemometrics, non-destructive measurement, biochemicals

## Abstract

This study investigated the effects of extraction methods on the physicochemical, phytochemical, and antioxidant properties of pomegranate juice (cv. Wonderful). In addition, the application of attenuated total reflectance Fourier transformed mid-infrared (ATR-FT-MIR) spectroscopy and chemometrics were explored in order to discriminate between different extraction methods. Juice variants evaluated included juice extracted without crushing the seeds (arils only) using a juice extractor (JE), juice extracted by crushing the seeds using a blender (arils plus seed) (JB), and juice extracted from half fruit using a commercial hand press juicer (CH). Juice extracted from CH had higher total soluble solid (TSS) content (18.20%), TSS/TA ratio (15.83), and color properties (a^*^ = 32.67, b^*^ = 11.80, C^*^ = 34.77) compared with extraction methods JE and JB. The juice extracted from JB showed the highest titratable acidity (2.17%), cloudiness (0.43), and lowest pH value (2.69). The total phenolics and anthocyanin content in the investigated juice ranged from 1.87 to 3.04 g gallic acid equivalent (GAE)/L and 37.74–43.67 mg cyanidin 3-glucoside equivalent/L of crude juice, respectively. Juice extracted from JB and CH was significantly higher in phenolic and anthocyanin compared with JE. Orthogonal partial least squares discriminant analysis (OPLS-DA) and principal component analysis (PCA) were used for classification. Classification accuracy of 100% was achieved between the three methods. The S-line plot revealed that the corresponding wavelength bands within the following regions 1,090, 1,250, 1,750, and 3,200 cm^−1^ were responsible for discrimination between the different extraction methods. Our results suggest that the main contributor to the discrimination between extraction methods were TSS, TSS/TA, color attributes, and anthocyanin content. Overall, this study has demonstrated that ATR-FT-MIR spectroscopy provides a powerful way to discriminate between juice extraction methods.

## Introduction

Pomegranate (*Punica granatum* L.) belongs to the Punicaceae family and has been classified as a superfruit in the global functional food industry. The fruit juice has received immense interest in research and consumption during the past 15 years due to the rich source of bioactive compounds when compared with other compound-rich beverages such as blueberry juice, cranberry juice, concord grape juice, red wine, and green tea (Seeram et al., 2008). These bioactive compounds have been mainly attributed to polyphenolic compounds such as phenolic acids, tannins, flavonols, and anthocyanins (Herceg et al., 2016; Kovačević et al., 2016; Putnik et al., 2019). Among the fruit parts, the nonedible portion (peel) has been described to contain the highest concentration of bioactive compounds such as ellagitannins (Mphahlele et al., 2016a). However, the seeds have been described to contain a unique source of unsaturated fatty acids and phenolic compounds (Seeram et al., 2006). Fresh pomegranate juice has been described to contain a diverse array of vitamins, mineral elements, and complex bioactive constituents. Thus, fresh pomegranate juice consumption offers a convenient and straightforward way to consume biologically active compounds compared with other parts of the fruit (Miguel et al., 2010; Viuda-Martos et al., 2010).

The desire of the consumers to maintain a diet that promotes better health has increased the demand for juices that preserve their natural nutritive value. However, the nutritive composition and bioactive properties in pomegranate juice are strongly influenced by microclimate, cultivar, maturity status, and extraction method (Rajasekar et al., 2012; Mditshwa et al., 2013; Mphahlele et al., 2018). Therefore, alternative processing methods that could potentially increase nutritive properties necessitate further investigation. Commercial production of pomegranate juice is mainly extracted from the fruit either by juicing the arils only, crushing the arils plus seed, or by compressing halved fruit/whole fruit that includes arils together with pith and membrane (Rajasekar et al., 2012; Mayuoni-Kirshenbaum et al., 2016; Mphahlele et al., 2018). Information obtained from the present study would clarify the efficacy of each extraction method and provide initial recommendations to the pomegranate industry on the associated characteristics resulting from the commercial production of pomegranate fruit juice.

Considering the unique health and sensory attributes of pomegranate juice, the ability to identify and quantify juice content in products is a desirable aspect among processors. Since it has a premium value, there is a risk for the potential adulteration of pomegranate juice with other types of juice varieties. Currently, chromatography coupled with mass spectroscopy is the most commonly used method for quality control, authentication, and detecting adulterated juice (Dasenaki and Thomaidis, 2019). However, these methods, though lengthy and require extensive extraction and purification steps, still remain the workhorses of high-end research applications. Contrary to the classical methods that are time-consuming, costly, and prone to produce waste, spectroscopic techniques are rapid, low-cost, non-destructive, and sensitive, and thus a preferable option for evaluating juice quality, authenticity, and adulteration (Jha and Gunasekaran, 2010; Shen et al., 2016; Arendse et al., 2020). Fourier transformed mid-infrared (FT-MIR) spectroscopy combined with chemometrics is arguably one of the most advanced non-destructive techniques used for evaluating the characteristics of juice quality and has been extensively applied in the classification of juices based on authenticity and adulteration (Vardin et al., 2008; Snyder et al., 2014; Arendse et al., 2020).

To our knowledge, this is the first application of the technique used for the classification of juice based on different extraction methods. This research will not only provide information on understanding the efficiency of different extraction methods on juice quality, but also allow the implementation of appropriate management strategies to classify pomegranate juice extracted through different methods. The aim of this study was 2-fold: the first was to evaluate the effect of different extraction methods on the quality and biochemical attributes of pomegranate juice, and the second was to investigate the application of Fourier transformed infrared (FT-IR) spectroscopy to discriminate between different extraction methods.

## Materials and Methods

### Fruit Supply and Processing

Pomegranate (cv. Wonderful) at commercial maturity was obtained from Sonlia commercial packhouse, located in the area of Western Cape Province, South Africa (33°34′851″S, 19°00′360″E). To add variability, fruit obtained from the packhouse was identified to originate from two different orchards (Wellington and Porterville). A total of 200 fruit was transported to the Postharvest Technology Research Laboratory at Stellenbosch University. On arrival, fruit was equilibrated at ambient conditions before being sorted out for physical defects and uniformity. Fruit was then stored at 7 ± 0.3°C, 91 ± 3% RH for <5 days before processing. Three different extraction methods were employed as demonstrated in Table 1. For each extraction method, a total of 60 fruit was processed. The extracted juice from each method was then immediately stored at −80°C until further analysis.

**Table 1:** Extraction techniques and fruit fractions used to obtain juice from pomegranate fruit (cv. Wonderful).

**Extraction method**	**Description**
Liqua fresh juice extractor (JE) (Mellerware. South Africa)	Juice was extracted from the arils by spinning at a minimum speed without crushing the seeds (kernels)
Electronic juice blender (JB) (AEG. Germany)	Arils were blended at a maximum speed using electronic blender for ~30s which ensured that the seeds were broken together with the arils
Commercial hand press juicer (CH) (Jupiter China)	Fruit halves were individually hand pressed. In this case, the pith, carpellary membrane, and arils were consistent during the extraction process

### Spectral Acquisition

Spectral acquisition of pomegranate juice was acquired in the mid-infrared region of 12,500–4,000 cm^−1^ using the Alpha-P ATR-FT-MIR spectrometer (Bruker Optics, Ettlingen, Germany) that measured diffuse reflectance. The Alpha-P spectrometer is equipped with a high-throughput ZnSe ATR diamond crystal, with six internal reflectors, and an exceptionally high light throughput providing the highest ATR measurement sensitivity for the analysis of even low concentrated sample components. Prior to obtaining FT-MIR spectra, reference measurements were performed against air background and periodically at intervals of 30 min during sample spectra acquisition. After every subsequent measurement, the scanning stage was cleaned using distilled water and tissue paper. The Alpha-P instrument was operated under the following conditions: sample plate temperature was maintained at 40°C, 4 cm^−1^ resolution scan, and 10 kHz scanner velocity with a total of 128 averaged background and sample scans per spectrum.

### Reference Measurements

#### Color Attributes

Juice color was assessed in CIELAB coordinates (L^*^, a^*^, b^*^) using a color meter (Chroma Meter, CR-400/410 Minolta Corp., Osaka, Japan) which were calibrated with a calibration white plate (*Y* = 85.2, *x* = 0.310, *y* = 0.3250). The color measurements were performed against a white background in a glass Petri dish with a measurement area of 5 cm. The L^*^, a^*^, and b^*^ values were used to calculate the color components, Chroma (C^*^), and hue angle (h°) as described by Pathare et al. (2013).

#### Juice Cloudiness and Density

In order to analyze the juice cloudiness, 5 ml of pomegranate juice was centrifuged at 12,000 × *g* for 10 min at 4°C. For cloudiness, the transmittance of the supernatant was measured at 650 nm using UV/visible spectrophotometer (Thermo Scientific Technologies, Madison, WI, USA) (Castagnini et al., 2015; Bora et al., 2017). Density of pomegranate juice was measured with an SG-Pro digital hydrometer.

#### Chemical Composition

A calibrated digital refractometer (Atago, Tokyo, Japan) was used to measure total soluble solids (TSS) and was expressed as percentage. A compact Metrohm titrosampler (Herisau, Switzerland) was used to determine the titratable acidity (TA) by titrating 2 ml juice against 0.1 N of NaOH solution to an end point of pH 8.2, and the results were expressed as a percentage of citric acid. The pH of the juice was evaluated at ambient temperature using a calibrated pH meter (Crison, Barcelona, Spain). The sugar-to-acidity ratio (TSS/TA) was also calculated. BrimA index, a variation of TSS and TA ratio and a criterion for the acceptance of juice, was assessed using TSS – *k* × TA, where *k* equals 2, thereby avoiding negative BrimA values (Jordan et al., 2001; Fawole and Opara, 2013).

### Sample Preparation for Phytochemical Analysis

Crude pomegranate juice (1 ml) was extracted with 29 ml of 50% aqueous methanol solution (v/v). The resulting mixture was vortexed and then sonicated (DC400H, MRC Ltd., Israel) (frequency: high setting and power: 0.05 W) for 20 min in a cold water bath followed by centrifuging at 10,000 rpm for 5 min at 4°C (Merk, Eppendorf AG, Germany). The supernatant was subsequently collected into clean vials and stored at −80°C until the assay for phenolic components and antioxidant properties was performed.

#### Determination of Total Phenolic Content

Total phenolic concentration (TPC) was determined by the Folin-Ciocalteu (Folin-C) colorimetric method as described by Makkar (2000) with modifications as reported by Fawole et al. (2012). In triplicate, supernatant (50 μl) was mixed with 450 μl of 50% methanol (v/v) before the addition of 500 μl of Folin C. After an incubation period of 5 min, 2.5 ml of 2% sodium carbonate (w/v) was added. The mixture was vortexed and, after 40 min, the absorbance was read at 725 nm against 50% blank aqueous methanol using a UV–visible spectrophotometer (Thermo Scientific Technologies, Madison, WI, USA). Final results were expressed as grams gallic acid equivalent (GAE) per liter of crude juice.

#### Determination of Total Anthocyanin Content

Quantification of total anthocyanin content (TAC) was performed as reported by Giusti and Wrolstad (2001) using the pH of differential methods. Briefly, 1 ml extracted supernatant was mixed with 9 ml of pH 1.0 (potassium chloride, 0.025 M) and pH 4.5 (sodium acetate, 0.4 M) buffer solution (pH 4.5), respectively. The absorbance was measured with a UV–visible spectrophotometer at 510 and 700 nm, respectively, and the results were expressed as cyanidin 3-glucoside using the following formula:

(1) $$\begin{array}{l} \text{A}={\left({\text{A}}_{520}-{\text{A}}_{700}\right)}_{\text{pH}1.0}-{\left({\text{A}}_{520}-{\text{A}}_{700}\right)}_{\text{pH}4.5} \end{array}$$

(2) $$\begin{array}{l} \text{Total anthocyanin}=\;\frac{A\;MW\;DF\;100}{\varepsilon \;L} \end{array}$$

where A is absorbance; MW is molecular weight (for cyanidin-3-glucoside 449.2 g mol^−1^); DF is dilution factor; *L* is cell path length (10 mm); and ε is the molar extinction coefficient (26,900). Final results were expressed as milligram cyanidin-3-glucoside equivalent per liter of crude juice.

#### Antioxidant Property

The radical scavenging ability of pomegranate juice was tested against the stable radical 2,2-diphenyl-l-picrylhydrazyl (DPPH). The DPPH assay was measured as described by Karioti et al. (2004) with modifications according to the study by Fawole et al. (2012). In duplicate, under dim light, 15 μl of the extracted sample was diluted with 735 μl of 100% methanol. This was followed by the addition of 750 μl methanolic DPPH solution. Samples were incubated for 30 min in darkness before the absorbance was measured at 517 nm with a UV–visible spectrophotometer. A stock solution of 1 mM ascorbic acid was prepared using 100% methanol (w/v) with a concentration ranging from 0.4 to 2.0 mM ascorbic acid. The results were expressed as micromole ascorbic acid (mg AA) equivalent per milliliter of crude pomegranate juice (mM AAE/ml).

Ferric ion reducing antioxidant power (FRAP) of pomegranate juice was measured calorimetrically as reported by Benzie and Strain (1996) with a slight modification (Fawole et al., 2012). The FRAP solution contained the following mixtures: 300 mM acetate buffer (50 ml), 10 mM 2,4,6-tripyridyl-striazine (TPTZ) (5 ml), and 20 mM ferric chloride (5 ml) which was incubated in a warm bath at 37°C before use. In triplicate, juice supernatant (150 μl) was added to 2,850 μl of the FRAP solution before incubation in darkness for 30 min before the absorbance was measured at 593 nm using a UV–visible spectrophotometer. A stock solution of 1 mM trolox was prepared using methanol (w/v) with a concentration ranging from 0.1 to 1.0 mM trolox. The results were expressed as trolox (mM) equivalents per milliliter pomegranate juice (mM TE/ml).

### Chemometric Analysis

Data processing and discriminant analysis were performed using SIMCA version 14 (Umetrics, Umeå, Sweden). In order to remove unwanted variation in the spectral data, several spectral filtering methods were evaluated. The baseline-corrected spectra were subjected to Savitzky–Golay transformation (first and second derivative), multiplicative scattering correction (MSC), and standard normal variate (SNV) correction. Explorative data analysis was performed using principal component analysis (PCA). PCA is an unsupervised method and is used to investigate the structure of a dataset and attempts to model the total variance of the original dataset *via* the uncorrelated principal components (Cozzolino et al., 2011). While orthogonal partial least squares discriminant analysis (OPLS-DA) is a supervised classification technique that isolates a predictive component and integrates an orthogonal correction filter, in order to differentiate the variation within the dataset (Wold et al., 1987; Bylesjö et al., 2006). Spectral data were subjected to PCA in order to investigate the separation of juice extraction methods and chemistry, while OPLS-DA was used for discrimination between different extraction methods.

Validation of the spectral dataset was accomplished by randomly splitting the dataset into test (50%) and train (50%) sets with the scale set as mean centering. OPLS-DA dataset was subjected to an S-Line plot. The S-line plot is especially suited for spectroscopy data. The plot displays the predictive loading in a form resembling the original spectra with the spectral peaks at the top end of the color scale influence the separation of the groups. Prediction datasets were subjected to variable importance projection (VIP), which summarizes the importance of the variables both to explain X and to correlate to Y. Terms with VIP values larger than 1 for variables show with large importance for model performance.

### Statistical Analysis

Statistical analyses were performed using statistical software (STATISTICA, version 16, StatSoft Inc., USA). Where appropriate, data were subjected to the one-way ANOVA according to Duncan's multiple range test and Pearson's correlation analysis. Results were presented as mean and standard errors for all studied variables.

## Results and Discussion

### Physicochemical Properties of Pomegranate Juice

Extraction method significantly (*p* < 0.05) affected the color properties of pomegranate juice (Table 2). Juice extracted by pressing half of the fruit using a commercial hand press (CH) showed the highest redness (a^*^ = 32.67), hue color (h° = 19.51), and color intensity (C^*^ = 34.77) compared with the rest of the extraction methods (Table 2). Our results are contrary to Miguel et al. (2004) who reported no significant differences in pomegranate juice color using two different extraction methods. Considering that each extraction method utilizes different fruit fractions, redness for pomegranate juice is in the order of half fruit > arils plus seed > arils. The higher red color readings obtained from CH extraction may be related to increased extractability of several anthocyanin and phenolic compounds found in the exposed rind, arils, and membrane of the halved pomegranate fruit.

**Table 2:** Color attributes of pomegranate juice (cv. Wonderful) extracted by using three different methods.

**Extraction method**	L*	a*	b*	**h^**°**^**	C*
Juice blender (JB)	19.27 ± 0.023c	18.00 ± 0.064c	2.57 ± 0.025c	7.73 ± 0.048c	18.23 ± 0.067c
Juice extractor (JE)	24.02 ± 0.050b	30.63 ± 0.073b	10.09 ± 0.064b	17.85 ± 0.068b	32.31 ± 0.089b
Commercial press (CH)	25.81 ± 0.051a	32.67 ± 0.078a	11.80 ± 0.064a	19.51 ± 0.06a	34.77 ± 0.094a

*Different letter(s) on column indicate statistical significant (p < 0.05) differences according to Duncan's multiple range test*.

For chemical properties, the method of extraction significantly (*p* < 0.05) influenced the TSS of pomegranate juice (Table 3). The juice extracted using the CH showed a higher TSS content (18.20%) compared with extraction using a juice blender (JB) (17.78%) and juice extractor (JE) (16.65%). A possible cause of low TSS content within JB and JE could be due to the crushing of arils which resulted in dilution due to the addition of seed content such as oil into the juice. In a similar study, Mphahlele et al. (2016b) reported higher individual soluble sugar and total sugar contents in juice extracted from half fruits compared with blending of the arils. However, the TA value (2.17%) was found to be significantly higher from juice extracted using the JB (arils plus seeds crushed) compared with the rest of the other extraction methods (Table 3). The TA value of 2.17% found in our study is higher than those reported by Mphahlele et al. (2016b) who found a lower TA value (1.56%) in juice from half pomegranates (cv. Wonderful). The ratio of TSS to TA value is an essential criterion for assessing the taste of pomegranate juice. The highest TSS/TA ratio was found in juice extracted from CH (Table 3). The TSS/TA ratio for pomegranate juice is in the order of CH (half fruit) > JB (arils plus seed) > JE (arils). In addition, we observed a strong negative correlation (−0.961) between TSS/TA ratio and TA value suggesting that TSS/TA ratio is highly influenced by TA content and is responsible for the bitter or sour taste in juice. This observation reported within our study is in agreement with those reported within literature (Mena et al., 2011; Arendse et al., 2015; Mphahlele et al., 2016b).

**Table 3:** Physicochemical properties of pomegranate juice (cv. Wonderful) extracted by using three different methods.

**Extraction method**	**TSS (%)**	**TA (%)**	**pH**	**TSS/TA**	**BrimA**	**Cloudiness@640 nm**	**Density (kg/m^**3**^)**
Juice blender (JB)	17.78 ± 0.11b	2.17 ± 0.089a	2.69 ± 0.023b	8.49 ± 0.032c	13.44 ± 0.022b	0.43 ± 0.017a	1.03 x 10^3^ ± 0.011a
Juice extractor (JE)	16.65 ± 0.14c	1.68 ± 0.074b	2.98 ± 0.033a	10.29 ± 0.059b	12.54 ± 0.11b	0.22 ± 0.011b	1.04 x 10^3^ ± 0.003a
Commercial press (CH)	18.20 ± 0.15a	1.19 ± 0.041c	3.02 ± 0.023a	15.83 ± 0.061a	15.83 ± 0.018a	0.18 ± 0.014c	1.04 x 10^3^ ± 0.003a

*TSS, total soluble solids; TA, titratable acidity. Different letter(s) on column indicate statistical significant (p < 0.05) differences according to Duncan's multiple range test*.

To further explore the relationship between TSS and TA as a potential maturity indicator, BrimA index was calculated according to the study by Jordan et al. (2001). Similar to the TSS/TA ratio, the highest BrimA index was observed in juice extracted from CH (15.83). A strong positive correlation (0.871) and negative correlation (−0.707) were observed between BrimA index, TSS, and TA, respectively. Strong correlations were found between TSS and BrimA, and this relationship suggests that BrimA is mainly influenced by TSS. The pH value of pomegranate juice characterizes its acidic taste, the former having an inverse correlation (−0.915) with the latter (Table 4). Pomegranate juice extracted from CH had a considerably higher pH value (3.02) than JE (2.98) and JB (2.69). Considering that pH values indicate the acidity of a solution, as bacterial growth is highly influenced by the pH of a medium, it could be suggested that juice extracted using a juice blender can be stored longer as low pH has the ability to inhibit bacterial growth. Similar range in pH values (2.50–2.66) using a blender (arils plus seed) has been reported by Rajasekar et al. (2012). Cloudiness of pomegranate juice was significantly affected by the extraction method. The JB method had the highest cloudiness value (0.43) compared with JE (0.22) and CH (0.18) (Table 3). The higher cloudiness observed in juice from the juice blender may be attributed to the breaking down of small particles from the seeds and arils, when they are crushed. Furthermore, a significantly negative correlation was observed with cloudiness and color attributes suggesting that an increase in juice cloudiness has an undesirable effect on the visual color of processed juice. For the density of pomegranate juice, no significant differences were observed between the three extraction methods (Table 3).

**Table 4:** Pearson correlation coefficient matrix between physicochemical, phytochemical, and antioxidant properties.

**Variables**	**TSS (%)**	**TA (%)**	**pH**	**TSS/TA ratio**	**BrimA**	L*	a*	b*	**Chroma**	**Hue**	**Density (kg/m3)**	**Cloudiness @ 650 nm**	**TPC (g/L)**	**TAC (mg/L)**	**DPPH (mM AAE/ml)**	**FRAP (mM trolox/ml)**
**TSS (%)**
TA (%)	−0.269															
pH	−0.143	−0.915														
TSS/TA ratio	0.526	−0.961	0.767													
BrimA	0.871	−0.707	0.361	0.876												
L*	0.012	−0.966	0.988	0.857	0.501											
a*	−0.126	−0.921	**1.000**	0.777	0.377	0.990										
b*	−0.080	−0.939	**0.998**	0.806	0.420	0.996	**0.999**									
Chroma	−0.116	−0.925	**1.000**	0.784	0.386	0.992	**1.000**	**0.999**								
Hue	−0.125	−0.922	**1.000**	0.778	0.379	0.991	**1.000**	**0.999**	**1.000**							
Density (kg/m3)	−0.300	−0.838	0.987	0.654	0.207	0.950	0.984	0.975	0.982	0.984						
Cloudiness	0.129	0.920	**−1.000**	−0.775	−0.374	−0.990	**−1.000**	**−0.999**	**−1.000**	**−1.000**	−0.985					
TPC (g/L)	0.715	0.481	−0.794	−0.219	0.279	−0.691	−0.784	−0.754	−0.778	−0.783	−0.881	0.786				
TAC (mg/L)	−0.525	0.961	−0.767	**−1.000**	−0.875	−0.858	−0.778	−0.807	−0.784	−0.779	−0.655	0.776	0.220			
DPPH (mM AAE/ml)	−0.702	−0.496	0.805	0.236	−0.263	0.703	0.795	0.765	0.789	0.794	0.890	−0.797	**0.997**	−0.237		
FRAP (mM trolox/ml)	**0.997**	−0.194	−0.219	0.459	0.831	−0.065	−0.202	−0.156	−0.192	−0.200	−0.372	0.205	0.766	0.458	0.755	

*Relationships with correlation coefficients >0.5 are presented. Correlation values in bold are significant at p < 0.05. TSS, total soluble solids; TA, titratable acidity; TPC, total phenolic content; TAC, total anthocyanin content; DPPH, 2,2-diphenyl-l-picrylhydrazyl; FRAP, ferric ion reducing antioxidant power*.

### Phytochemical and Antioxidant Properties of Pomegranate Juice

Methods of extraction significantly (*p* < 0.05) influence the total phenolic concentration of the pomegranate juice (Table 5). Juice extracted from the JB had the highest phenolic concentration (3.04 g/L) than the rest of the extraction methods (Table 5). Total phenolic concentration is in the order of JB (3.04 GAE g/L) > CH (2.47 GAE g/L) > JE (1.87 GAE g/L). We presumed that the high phenolic concentration in JB may be attributed to the extraction of phenolic compounds from the arils and seeds. Contrary to our results, Mphahlele et al. (2016a) reported a higher total phenolic concentration in juice extracted from half fruit compared with crushing of the arils. Anthocyanin compounds are responsible for the desirable red color of pomegranate juices as well as many other red-colored fruit juices (Li et al., 2010; Fischer et al., 2013). In this study, pomegranate juice from all extraction methods showed abundant of total anthocyanin content. We observed significantly (*p* < 0.05) higher levels of anthocyanin in juice extracted using JB (43.67 mg/L). Kalt et al. (2000) observed higher levels of anthocyanin at a low pH level, which is consistent with the presence of the intensely colored flavylium cations compared with the quinonoidal pseudobase, and chalcone forms, which are pale or colorless. Therefore, it could be argued that the high anthocyanin content observed from the JB juice resulted from a low pH (2.69) contained within the juice. Another interesting relationship was the high negative correlation between anthocyanin concentration and color components (a^*^ = −0.778, b^*^ = −0.807, C^*^ = −0.784, h° = −0.779) (Table 4). This negative correlation between color components and color pigment anthocyanin may suggest that anthocyanin could be used as an indicator of juice color. A strong negative correlation between anthocyanin and raspberry juice was observed by Palonena and Weber (2019).

**Table 5:** Phytochemical and antioxidant properties of pomegranate juice (cv. Wonderful) extracted by using three different methods.

**Extraction method**	**TPC (g/L)**	**TAC (mg/L)**	**DPPH (mM AAE/ml)**	**FRAP (mM trolox/ml)**
Juice blender (JB)	3.04 ± 0.012a	43.67 ± 0.12a	0.16 ± 0.0012c	0.57 ± 0.08a
Juice extractor (JE)	1.87 ± 0.017c	39.73 ± 0.20b	0.29 ± 0.0012a	0.53 ± 0.021b
Commercial press (CH)	2.47 ± 0.011b	37.74 ± 0.12b	0.22 ± 0.0014b	0.58 ± 0.02a

*TPC, total phenolic content; TAC, total anthocyanin content; DPPH, 2,2-diphenyl-l-picrylhydrazyl; FRAP, ferric ion reducing antioxidant power. Different letter(s) on column indicate statistical significant (p < 0.05) differences according to Duncan's multiple range test*.

Significantly higher (*p* < 0.05) antioxidant activity measured by radical scavenging activity was found from juice extracted using the JE (0.29 mM AAE/ml), whereas the JB (0.16 mM AAE/ml) had the least (Table 5). Non-significant but strong correlation was observed between DPPH assay and total phenolic concentration (0.997), suggesting that the concentration of phenolic compound plays a role in the antioxidant properties of pomegranate juice (Bunea et al., 2011) (Table 4). A similar comparative study of homemade and commercial grape juice by Burin et al. (2010) reports a correlation coefficient of 0.957 between total phenolic concentration and DPPH antioxidant activity. FRAP assay measures the antioxidant potential in juice by reducing ferric iron (Fe^3+^) to ferrous iron (Fe^2+^) by antioxidants. In this study, JB (0.57 ± 0.02 mM trolox/ml) and CH (0.58 ± 0.02 mM trolox/ml) extraction method did not vary significantly (*p* > 0.05) in antioxidant activity (Table 5). The higher FRAP antioxidant activity was observed in both JB and CH, and this may be attributed to the effective extraction of phenolic compounds from the seeds of the arils or peel of the fruit during the extraction process. Furthermore, a high correlation was observed between FRAP and total phenolic concentration (0.776), suggesting that phenolic compound concentration plays a role in the antioxidant potential of pomegranate juice. Previous studies observed high correlations between juice phenolics and antioxidant capacity (Gil et al., 2000; Shwartz et al., 2009), evidently suggesting that phenolic compounds are the main antioxidants in pomegranate juice.

### Fourier Transformed Infrared Spectral Characterization of Pomegranate Juice Using PCA

**Figure 1:**
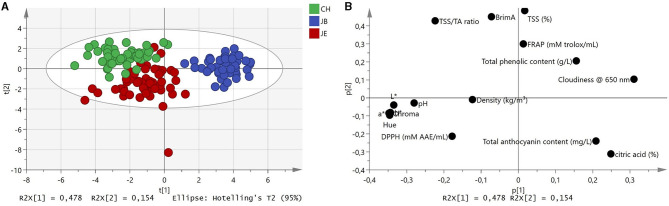
**(A)** Principal component analysis score plot of spectral data and **(B)** score plot with associated chemistry. Green, commercial hand press juicer (CH); blue, juice blender (JB) (crushing the arils with the seeds); and red, juice extractor (JE) (extract juice from arils without crushing the seeds).

Preliminary assessment of MIR spectra was investigated using PCA to examine the effects of the extraction method on pomegranate juice quality. The application of several spectral filters such as Savitzky–Golay first derivative, SNV, and MSC did not improve the discrimination of the PCA scores plot. The examination of score plots from baseline-corrected spectra showed the effects of extraction on the spectral and chemical properties of pomegranate juice (Figure 1). By plotting all 150 data points, scores from the first two PC explained 63.2% (PC_1_ = 47.8%, PC_2_ = 15.4%) of the total variation within the dataset (Figure 1A). Examination of the PCA scores plot generated from the three extraction methods showed well-defined sample clusters, whereas the CH and JE extraction methods co-clustered with one another (Figure 1A). Furthermore, the PCA plot may have revealed possible grouping (JB) of the spectral and chemical dataset (Figure 1B). For instance, a high level of juice cloudiness was observed for JB extraction (Table 3). This observation showed that IR spectroscopy could be used to differentiate between different extraction methods based on spectral and chemical data despite its simplified approach. Therefore, to examine the effects of the extraction method on pomegranate juice quality, the spectral and chemical datasets were further subjected to OPLS-DA.

**Figure 2:**
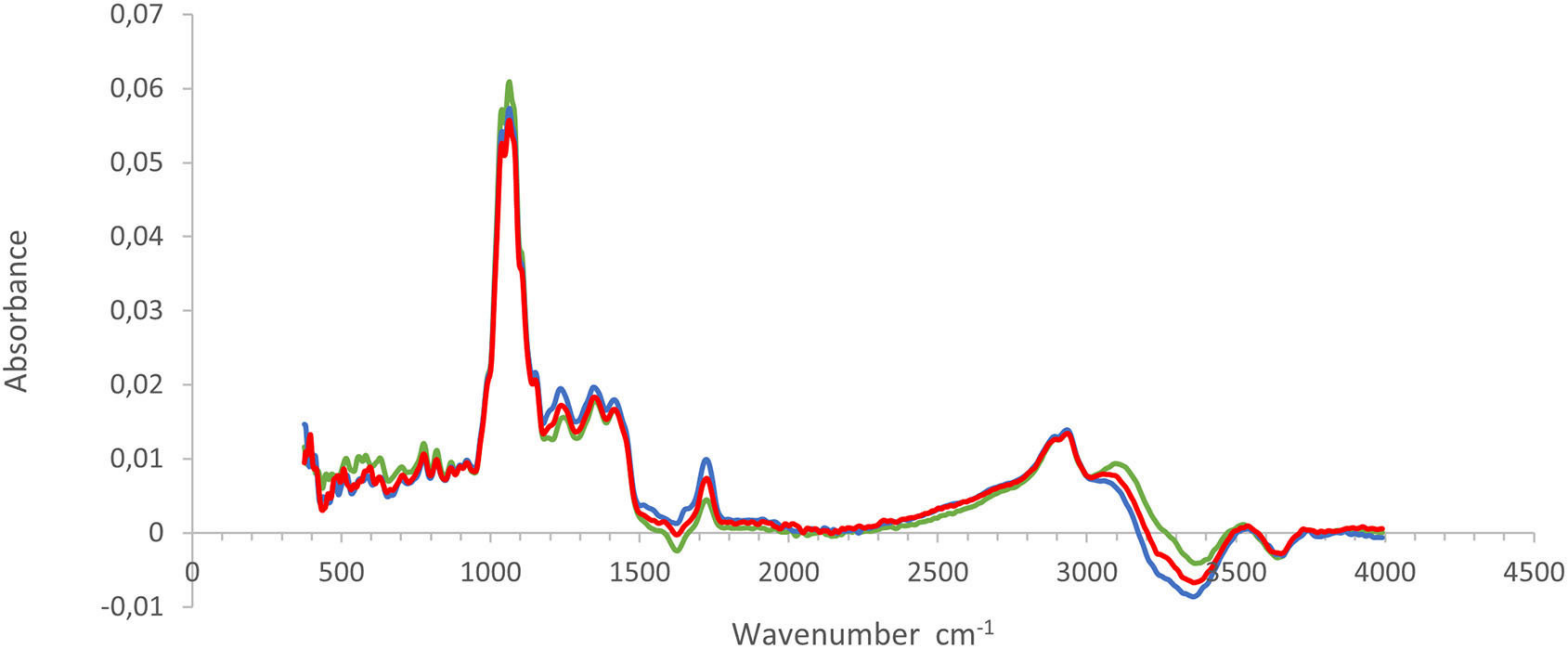
Description of the FT-IR spectral characteristics from pomegranate juice extracted by using three different methods. Green, commercial hand press juicer (CH); blue, juice blender (JB) (crushing the arils with the seeds); and red, juice extractor (JE) (extract juice from arils without crushing the seeds).

The average baseline-corrected absorbance spectra for pomegranate juice extracted from three different methods are shown in Figure 2. The MIR absorbance spectra for all three extraction methods showed similar contours and wavelength bands. Assessment of wave bands was performed according to literature (Bureau et al., 2019). The spectral bands displayed contours having prominent absorbance peaks in the following regions: 1,062, 1,150, 1,350, 1,420, 1,726, 2,940, 3,070, and 3,526 cm^−1^. The prominent bands observed at the regions of 1,726 cm^−1^ and 3,526–3,070 cm^−1^ have known to correspond to the O–H stretch in the water. The characteristic band observed at 2,940 cm^−1^ (corresponding to C–H_3_) is indicative of the presence of color pigments, such as chlorophyll and flavonoids, as interpreted by Favaro et al. (2018). The IR region between 1,420 and 950 cm^−1^ is usually referred to as the “fingerprint” region, as previously reported by Nieuwoudt et al. (2004). This region corresponds to the vibration of the C–O, C–C, C–H, and C–N bonds and provides information regarding organic compounds such as sugars, alcohols, and organic acids that are probably present in a sample (Favaro et al., 2018). The bands observed at 1,420–1,150 cm^−1^ have been ascribed to C–H, O–H, and C–O functional groups that are present in organic acids, while the most intense bands located in the region of 1,000–1,100 cm^−1^ have been reported to be due to the contribution of the C–O stretch related to carbohydrates emanating from sugars (Moreira and Santos, 2004; Musingarabwi et al., 2016). The spectral profile for pomegranate juice is similar to those reported by several researchers who evaluated the quality characteristics of apple/cranberry juice (Samborska et al., 2018), orange juice (Clark, 2016) and peach nectar (Coelho et al., 2020).

### Discrimination of Pomegranate Juice Based on FT-IR Spectra Using OPLS-DA

OPLSA-DA was applied on two different extraction methods at a time (pairwise), and the discrimination of these different methods was based on the spectral wave numbers and the associated chemistry (Supplementary Figures). By comparing the JB and JE, OPLS-DA was capable of providing 100% discrimination between these two methods (Supplementary Figure 1A). From pairwise OPLS-DA, the S-line plot revealed that the wave numbers responsible for discrimination between the methods were within the following regions: 3,201, 1,750–1,091 cm^−1^ (Supplementary Figure 1B). These wave numbers have been associated with O–H bonds of water molecules and C=O, C–O, C–C bonds of various compounds (carbohydrates, organic acids, flavonoids) as interpreted by several authors (Sinelli et al., 2008; Arendse et al., 2018; Favaro et al., 2018). The PCA score plot revealed that the extraction method JE is associated with pH and various color attributes (C^*^, a^*^, b^*^, h°), while the extraction method JB has been associated with TSS, cloudiness, and total phenolic content (Supplementary Figure 1C). The VIP plot for the chemistry suggests that color attributes and cloudiness contribute more to the separation of JB and JE extraction methods (Supplementary Figure 1D).

Likewise, by comparing the extraction method JB and CH, OPLS-DA was capable of providing 100% discrimination (Supplementary Figure 2A). The S-line plot (Supplementary Figure 2B) showed wavelength bands within the region of 3,200, 1,750, 1,691, 1,250, and 1,090 cm^−1^. The predominant band in the region of 1,090 cm^−1^ can be assigned to the C–O stretching vibration of the sugars. Similar bands in the region of 1,000–1,090 cm^−1^ were reported by He et al. (2007), for the evaluation of sugars in different commercial juices. The VIP plot suggests that color attributes, TSS/TA ratio, cloudiness, and anthocyanin contribute toward the separation of these two methods (Supplementary Figure 2D). The pairwise comparison between CH and JE resulted in 100% classification between the two methods using OPLS-DA (Supplementary Figure 3A). From the pairwise OPLS-DA, the S-line plot revealed that the MIR wave numbers responsible for discrimination between CH and JE were similar to those observed in Supplementary Figure 2B. The PCA score plot revealed that the extraction method CH is associated with TSS, BrimA, and TSS/TA ratio, while the extraction method JE has been associated with TA, total anthocyanin content, and total phenolic content (Supplementary Figure 3C). The main contributors to the separation of the two extraction methods were TSS/TA ratio, TA, TSS, total anthocyanin concentration, and color attributes (Supplementary Figure 3D). Noticeably, a high correlation was observed between various color attributes and anthocyanin concentration within this study, suggesting that FT-IR spectroscopy can distinguish juice processing methods based on flavonoid color pigments, such as anthocyanins.

**Figure 3:**
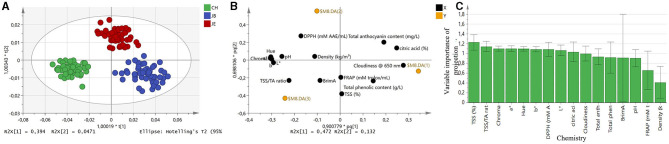
**(A)** The orthogonal partial least squares discriminant analysis score plot for all spectra *n* = 150. **(B)** Score plot representing extraction methods and associated chemistry. **(C)** Variable importance of projection (VIP) summarizing reference data is responsible for model performance. Color coded based on the type of extraction method. Green, commercial hand press juicer (CH); blue, juice blender (JB) (crushing the arils with the seeds); and red, juice extractor (JE) (extract juice from arils without crushing the seeds).

To further investigate the discriminating power of FT-IR spectroscopy, OPLS-DA was applied to all three different extraction methods, including the CH, JB, and JE. OPLS-DA correctly classified all three extraction methods with 100% accuracy (Figure 3A). The score plot representing all three extraction methods suggests that CH has been associated with the chemistry of TSS/TA ratio and BrimA (Figure 3B). The extraction method JE has been associated with total anthocyanin content suggesting higher red color, while JB has been observed to be associated with cloudiness and total phenolic content (Figure 3B). The VIP plot summarizing the chemistry responsible for model performance is shown in Figure 3C. When comparing all three methods, the VIP plot suggests that TSS contributes slightly more to separate the three extraction methods. Similarly, to the pairwise comparison, the VIP plot also illustrates that TSS/TA ratio and color components (L^*^, a^*^, C^*^, h°) all contribute toward model performance. The results of this study suggest that juice extraction methods can clearly be distinguished from one another based on TSS, acidity, anthocyanin concentration, color attributes, and flavor-associated compounds.

## Conclusion

This study has demonstrated that juice processing methods affect the physicochemical, phytochemical, and antioxidant properties of pomegranate juice. When utilizing different parts of the fruit (e.g., seeds, membrane, and peel) during the extraction process, we observed higher color attributes and phytochemical and antioxidant properties. This can be attributed to the effective extraction of phenolic compounds from the peel and membrane of fruit during the extraction process. Therefore, juice should be extracted from whole or half fruit to improve the organoleptic and antioxidant properties. Classification of pomegranate juice was performed according to the processing method using ATR-FT-MIR spectroscopy. Nondestructive discrimination between the juice processing methods was accomplished using PCA and OPLS-DA. Classification accuracy of 100% was achieved between the three methods. The S-line plot revealed that some of the most important wave bands within the following regions (1,090, 1,250, 1,750, and 3,200 cm^−1^) are responsible for discrimination between the different extraction methods. Our results suggest that the discrimination of main contributor of the extraction methods was TSS, acidity and its ratio, color attributes, and anthocyanin concentration. For juice processors in the food and beverage industry, this study first provides relevant information on existing juice extraction methods that may improve the sensory attributes of pomegranate juice during processing. Second, the developed method using an ATR-FT-MIR spectroscopy can be implemented in controlled processes for online and inline grading of pomegranate juice.

## Data Availability Statement

The original contributions presented in the study are included in the article/Supplementary Material, further inquiries can be directed to the corresponding author/s.

## Author Contributions

UO: conceptualization, methodology, funding acquisition, project administration, supervision, review, and editing. HN: methodology, software, and validation. OF: review and editing. EA: investigation, data capturing, formal analysis, writing, and original draft preparation. All authors have read and agreed to the published version of the manuscript.

## Author Disclaimer

The opinions, findings, and conclusions or recommendations expressed are those of the author(s) alone, and the NRF accepts no liability whatsoever in this regard.

## Conflict of Interest

The authors declare that the research was conducted in the absence of any commercial or financial relationships that could be construed as a potential conflict of interest.

## Publisher's Note

All claims expressed in this article are solely those of the authors and do not necessarily represent those of their affiliated organizations, or those of the publisher, the editors and the reviewers. Any product that may be evaluated in this article, or claim that may be made by its manufacturer, is not guaranteed or endorsed by the publisher.
