# Supplementary material for: Effect of Different Extraction Methods on the Quality and Biochemical Attributes of Pomegranate Juice and the Application of Fourier Transformed Infrared Spectroscopy in Discriminating Between Different Extraction Methods
Source: Front Plant Sci. 2021 Aug 23;12:702575. doi: 10.3389/fpls.2021.702575 (PMC8419332; doi:10.3389/fpls.2021.702575)
Supplement: Supplementary file 1 [file Data_Sheet_1.PDF]

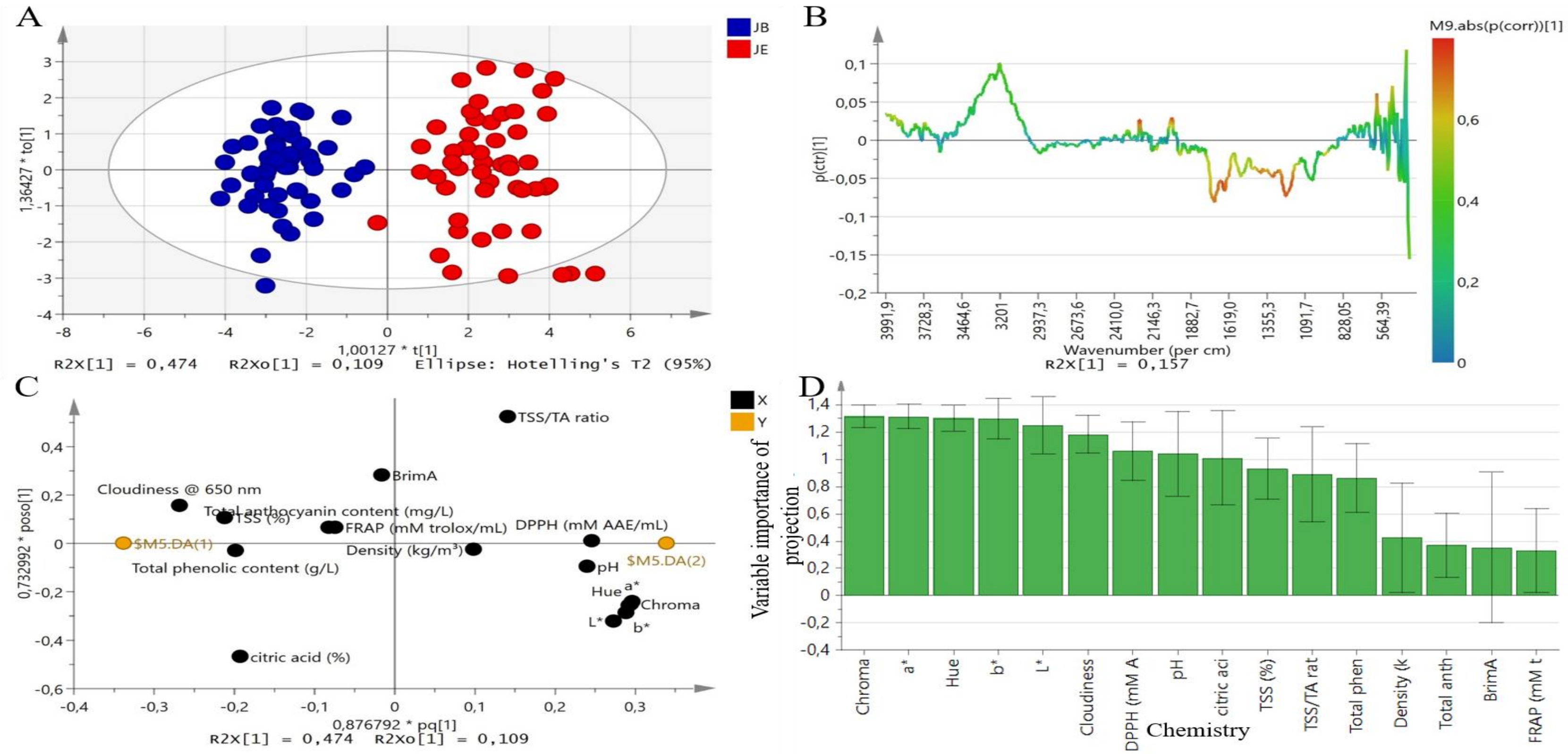

.

Supplementary Figure 1. Supplementary Figure 1. OPLS-DA score plot for all spectra n = 100, JB = juice blender and JE = juice extractor (A), the S-line plot showing the wavenumbers responsible for discrimination between the two extraction methods (B), score plot representing extraction methods and associated chemistry (C), variable importance of projection summarizing the important reference methods responsible for models performance.

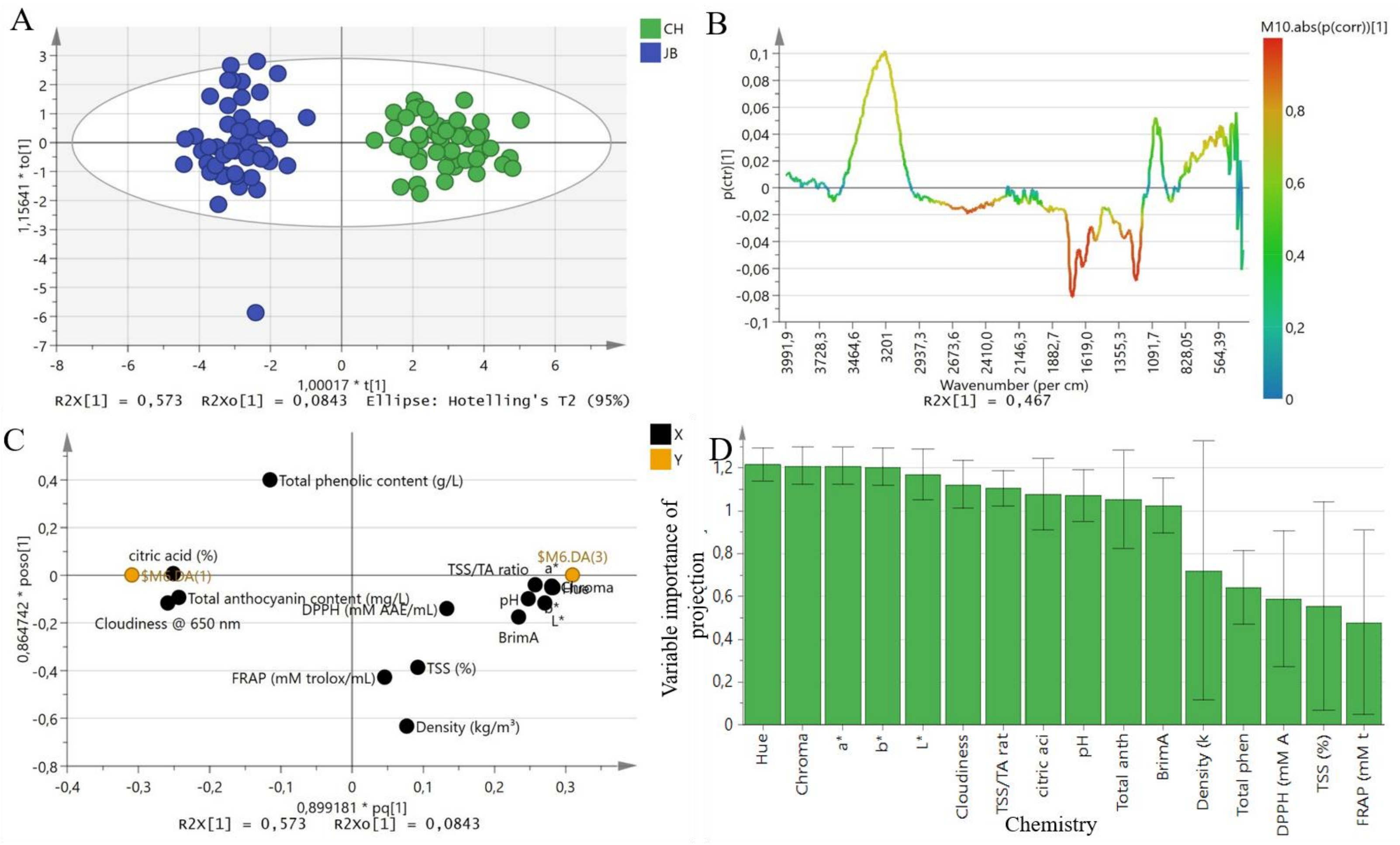

Supplementary Figure 2. OPLS-DA score plot for all spectra n = 100, JB = juice blender and CH = mechanical hand press (A), the S-line plot showing the wavenumbers responsible for discrimination between the two extraction methods (B), score plot representing extraction methods and associated chemistry (C), variable importance of projection summarizing the important reference methods responsible for models performance.

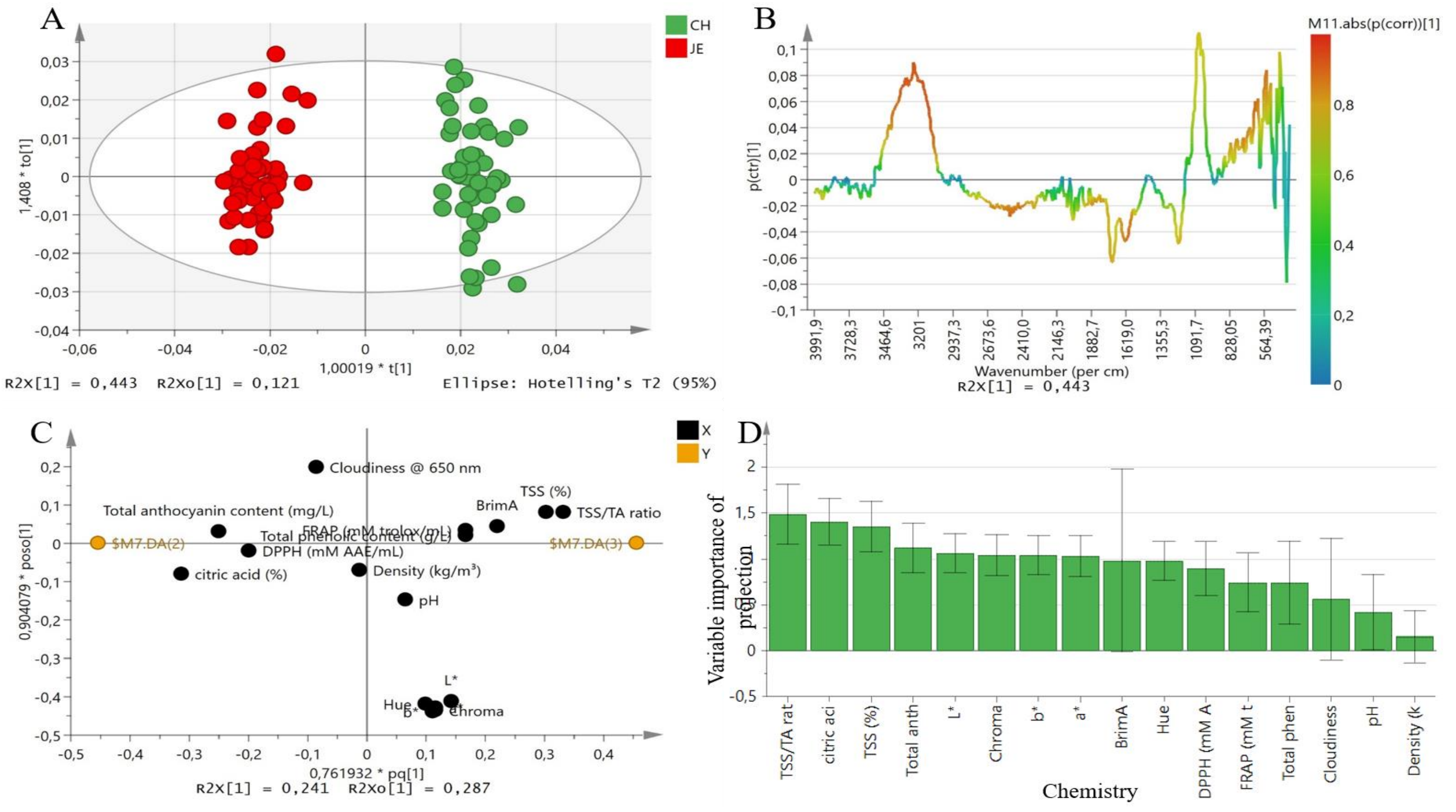

Supplementary Figure 3. OPLS-DA score plot for all spectra n = 100, CH = mechanical hand press and JE = juice extractor (A), the S-line plot showing the wavenumbers responsible for discrimination between the two extraction methods (B), score plot representing extraction methods and associated chemistry (C), variable importance of projection summarizing the important reference methods responsible for models performance
